# Obscure Case

**Published:** 1872-12

**Authors:** H. Scott


### Obscure Case.

BY H. SCOTT.

A young man of twenty years came with his mother to have his teeth filled. The first one he wished filled was one that was aching. He pointed out the second left inferior bicuspid. It had ached "*coming along*," he said. It was entirely sound in every respect, except the smallest decay on the grinding surface.

His upper incisors were more or less decayed, the left lateral considerably. I examined them with probe; and when I had passed it down into the left lateral, firmly, the lower bicuspid began to ache so severely as to bring tears from his eyes, and cause him to writhe in his seat. Creosote applied to the incisors brought instant relief to the bicuspid. I subsequently devitalized the upper nerve, and that was the last of the case.

Such cases are of course common to the profession, and I only communciate this to admonish those of less experience to be careful of diagnosis before acting in obscure cases. A fatal error would have been the extraction of the tooth the man complained of; a thing that is often done by the inexperienced.

